# Neighborhood farm density, types of agriculture, and depressive symptoms among older farmers: a cross-sectional study

**DOI:** 10.1186/s12889-021-10469-6

**Published:** 2021-03-04

**Authors:** Mariko Kanamori, Masamichi Hanazato, Katsunori Kondo, Andrew Stickley, Naoki Kondo

**Affiliations:** 1 Department of Health and Social Behavior, Faculty of Medicine, The University of Tokyo, Bldg. 3, 7-3-1 Hongo, Bunkyo-ku, Tokyo, Japan; 2 Department of Social Epidemiology, Graduate School of Medicine, Kyoto University, Floor 2, Science Frontier Laboratory, Yoshida-konoe-cho, Sakyo-ku, Kyotoshi, Kyoto, Japan; 3 Department of Social Preventive Medical Sciences, Center for Preventive Medical Sciences, Chiba University, 6 Chome-2, Kashiwanoha, Kashiwa, Chiba, Japan; 4 Department of Gerontological Evaluation, Center for Gerontology and Social Science, National Center for Geriatrics and Gerontology, 7 Chome 430, Moriokacho, Obu, Aichi Japan; 5 Department of Preventive Intervention for Psychiatric Disorders, National Institute of Mental Health, National Center of Neurology and Psychiatry, 4-1-1 Ogawa-Higashi, Kodaira, Tokyo, Japan

**Keywords:** Farm density, Depression, Farmer, Neighborhood, Japan, Older adults, Animal husbandry, Crop, Agriculture, Health effect

## Abstract

**Background:**

Farmers may have an increased risk for poor mental health. In connection with this, factors specific to the neighborhood environment such as farm density and the type of agriculture, might be important for mental wellbeing. In this study we aimed to clarify the cross-level interaction on depressive symptoms between farm density at the neighborhood level by type of agriculture and the longest occupation of individuals (farmer or non-farmer).

**Methods:**

Data came from the 2016 wave of the Japan Gerontological Evaluation Study (JAGES) that were linked to governmental agricultural data. Information was analyzed from 147,549 respondents aged 65 years or older, residing in 1024 neighborhoods in 39 municipalities. We calculated farm (crop or animal husbandry) density at the neighborhood level, dividing the number of agricultural management entities by the population. Three-level (individual, neighborhood, and municipality) Poisson regression analysis was used to calculate the prevalence rate ratios of depressive symptoms.

**Results:**

The prevalence of depressive symptoms was higher among individuals whose longest occupation was farmer compared to non-farmer. The estimated probability of depressive symptoms by a cross-level interaction analysis showed that among farmers of both genders, those who were residing in neighborhoods where the farm density was low had a higher prevalence of depressive symptoms, regardless of the type of agriculture. The slope of the relationship between depressive symptoms and animal husbandry farm density varied by occupation, with a higher prevalence of depressive symptoms observed in male farmers compared to male non-farmers.

**Conclusions:**

The high prevalence of depressive symptoms among farmers in neighborhoods with a low farm density may reflect a scarcity of formal and informal social support in such communities. The health effects of the neighborhood environment on farmers, such as farm density, which may vary by the type of agriculture, should be further researched.

## Background

Farmers are one of the groups that have been reported to have poor mental health worldwide, which suggests that structural factors in agriculture may play a role in their psychological wellbeing. In various countries, a variety of mental health outcomes including depression, anxiety, and psychological morbidity are all higher among farmers compared to non-farmers [1, 2]. Systematic reviews on suicide and occupation have also found that the suicide risk among farmers is higher than among individuals in other jobs [3, 4]. Although farming practices and the agricultural context vary among farms within and between countries, there is a common global context including increasing global competition, a decline in the number of farms, increasing farm size, and climate variability [3, 5–7]. The structural changes that are occurring in the agricultural sector are linked to a number of risk factors for poor mental health among farmers including an increasing economic burden, geographical and social isolation, as well as difficulty in obtaining help [1, 3, 8, 9].

In Japan, farming is characterized by both a declining and aging farming population; between 2000 and 2015 the number of people who worked mainly as farmers fell from 2.4 to 1.8 million, while in the same period the proportion of farmers aged 65 or above rose from 51 to 65% [10]. As the number of Japanese farms has decreased, it is possible that many farmers may have lost both friends and colleagues as other farmers have left farming communities. Losing this potential source of social support and social participation might have had a detrimental impact on farmers’ mental health [9, 11]. However, to our knowledge, as yet, no study has investigated the association between agricultural structural change, as reflected for instance, in neighborhood farm density, and farmers’ mental health, despite some evidence that Japanese farmers might be at an increased risk for worse mental health outcomes such as suicide [3, 12–14].

Different types of agriculture may be associated with differing community characteristics, which could also potentially relate to mental health problems. For example, studies have shown that work-related factors pertaining to animal husbandry, including a lack of off-season, animal diseases, economic pressure, as well as exposure to the death of livestock may all impact on a farmer’s mental health [15–18]. Indeed, a recent ecological study that used Japanese municipal data found that suicide mortality was positively associated with animal husbandry output per unit of the municipality population whereas no association was observed for other forms of agricultural output [19]. However, that study did not take individual characteristics such as occupation into consideration and did not determine whether farmers had a higher suicide risk compared to other (non-farming) residents. This may have been an important omission as there is some evidence that community characteristics relating to the type of agriculture may affect not only farmers, but rather, *all* people in the community [20, 21].

With this in mind, this study had two objectives. First, using individual data from older adults in Japan, we aimed to determine if there was a cross-level interaction for depressive symptoms between farm density at the neighborhood level (animal husbandry or crop farming) and occupation (farmer or non-farmer). Second, we tested if the prevalence of depressive symptoms among residents was higher in areas where animal husbandry was common compared to areas where it was less common, and whether the association differed depending on the main occupation of the residents (farmer or non-farmer).

## Methods

### Data

Cross-sectional data were drawn from the 2016 wave of the Japan Gerontological Evaluation Study (JAGES). The JAGES project conducted an anonymous self-administered mail survey, in cooperation with municipalities across Japan. More specifically, a questionnaire was sent to residents aged 65 years or older in participating municipalities, who were not certified as needing public long-term care insurance. The 2016 wave included individuals living in 39 municipalities in Japan. Details of the participating municipalities are available on the JAGES website [22]. In 22 generally larger municipalities, participants were selected by multistage random sampling, while in 17 smaller municipalities all eligible individuals were selected. In total 279,661 questionnaires were mailed, of which 196,438 were returned (response rate = 70.2%). After excluding those who were certified as needing public long-term care insurance, data were available for 180,021 individuals. The data has a three-stage hierarchical structure; individuals, school districts (neighborhoods), and municipalities.

To evaluate what types of agriculture were present in each residential area we obtained government agricultural statistics for 2015 from the website of the Ministry of Agriculture, Forestry and Fisheries [23]. We used them to proportionally distribute agricultural data into each JAGES school district. We also obtained government geographical data relating to the covariates used in this study.

### Measurements

#### Outcome: depressive symptoms

The Japanese short version of the Geriatric Depression Scale (GDS-15) was used to evaluate depressive symptoms. This measure was developed to assess depressive symptoms in self-administered surveys and consists of 15 items with yes (scored 1) and no (0) answer options that give a total score that can range from 0 to 15 where higher scores indicate more depressive symptoms. We used a cut-off score of 6 and above to categorize depressive symptoms, as this score was used to indicate moderate depressive symptoms in an earlier validation study among older Japanese adults [24]. This cut-off score has also been reported to be highly associated with suicidal ideation [25].

#### Farm density by type(s) of agriculture

We calculated farm density at the school district level, i.e. the number of farms (agricultural management entities) per unit of the total population. First, we aggregated the data by the type of agriculture into “animal husbandry” and “crop” categories. The animal husbandry category included dairy farming, beef cattle farming, pig farming, poultry farming, sericulture, and other livestock activities. The crop category included the production of rice, wheat, potatoes, craft crops, vegetables grown outdoors, vegetables grown in greenhouses, fruits, flowers, and other agricultural crops. Next, we divided the number of agricultural management entities by unit total population. As the distribution of these values was right-skewed and contained 0, we added 0.5 to each value and logarithmically transformed them. Then, we standardized these values (subtracted the average from each figure and divided it by the standard deviation).

#### Longest occupation

Although many individuals can be categorized as engaging in ‘farming’ in Japan, including those who start small-scale farming following their retirement, in this study we defined a farmer as anyone who reported that the longest occupation they had engaged in across their life course was farming. This was done as the principal aim of this study was to determine the health effects of demographic and structural change in agriculture. We gathered information on each respondent’s longest occupation by asking, “What is the type of occupation that you have participated in for the longest period in your life?” There were 9 predetermined response categories including (1) professional/technical, (2) managerial, (3) clerical, (4) sales/service, (5) skilled labor, (6) agriculture, forestry or fisheries, (7) self-employment other than agriculture, forestry, and fisheries, (8) other, (9) “I have never had a job.” Respondents who answered ‘agriculture, forestry or fisheries’ were then classified as being “farmers”, while all others were categorized as being “non-farmers.”

#### Covariates

The development of agriculture is affected by geographical and climate factors. Using the literature that has examined the association between geographical and climatic factors and suicide as a guide [26–28], we included the following variables as potential confounding factors in the association between farm density by type of agriculture and depression: length of daylight hours (classified in tertiles - short, middle, long), amount of rainfall, and the mean slope angle of the habitable land, which were all calculated at the neighborhood level. Covariates relating to individual socioeconomic status included years of education (less than nine years or not i.e. equivalent to graduating junior high school), equivalized household income tertile (high, middle, low), marital status (having a spouse or not), and living alone or not [29–33]. We also adjusted by population density quintile (highest, high, middle, low, lowest) at the neighborhood level [34], by the administrative regions in Japan (Tokai, Hokkaido, Tohoku, Hokuriku, Kanto and Higashiyama, Kinki, Kyushu) using area dummies, and age (65–74, 75–84, > = 85).

### Statistical analyses

In total 32,472 respondents were excluded from the analysis, including those without valid values for age (*n* = 262), gender (*n* = 30), residential address (*n* = 7), and depressive symptoms (*n* = 32,173). We calculated the prevalence ratio (PRs) of depressive symptoms using three-level (individual, neighborhood, and municipality) Poisson regression to take into account the hierarchical structure of the data containing variables measured at different areal levels. In order to avoid overestimating the PRs, Poisson regression with robust variance was used to model depressive symptoms, which can be regarded as a frequently occurring outcome [35]. We stratified all analyses by gender as mental health problems among farmers may be associated with socially constructed gender relations [8, 36]. We used the *mepoisson* command in STATA/MP 15.1 and incorporated random intercepts at the neighborhood level and the municipality level (StataCorp, LLC, College Station, Texas, USA). As we could not find a program to conduct multiple imputation using a three-level model, we created dummy variables for the missing covariate data and modeled them. We first analyzed a null (empty) model to evaluate the variance of the random parameters. To evaluate general contextual effects, we calculated Median Rate Ratios (MRRs) from the estimated variance of the random parameters. MRR refers to the median relative difference in the prevalence of depressive symptoms when comparing two randomly selected residents from groups with a higher and a lower prevalence of depressive symptoms [37]. We evaluated cross-level interaction effects, i.e. “whether the nature of a lower-level relationship depends on a higher-level factor” [38]. To do so, we included higher-level explanatory variables (animal husbandry farm density and crop farm density), lower-level variables (longest occupation), an interaction term between each type of farm density (Model 1: animal husbandry farm density; Model 2: crop farm density) and longest occupation, and all the covariates in the model. From these models, we obtained the estimated probability of outcomes at the farm density quantile (lowest, low, high, highest). As there were many cases where the value of animal husbandry farm density was 0, we combined the first and second percentile of the animal husbandry farm density variable and then obtained estimates (low, middle, high). Furthermore, to evaluate whether the variation in depressive risk according to farm density differed by longest occupation, we evaluated the statistical significance of the cross-level interaction term. As the statistical power of cross-level interaction tests is severely limited, we considered a value of *p* < 0.20 as being indicative of a potentially significant interaction effect [38, 39].

## Results

Data were analyzed from 70,988 men and 76,561 women, nested within 1024 neighborhoods in 39 municipalities. As shown in Table 1, about 4% of all respondents classified their longest occupation as farmer. More participants lived in the Tokai region than in any other region. Descriptive data of the study sample with the prevalence of depressive symptoms showed that 17.1% of all participants had depressive symptoms, and that the prevalence of depression was higher among farmers than non-farmers.

**Table 1:** Descriptive statistics of the study participants with the prevalence of depressive symptoms

	Total	Men	Women
	Number of participants (prevalence of depressive symptoms (%))		
*Individual-level variables*			
**Age**			
65–74	86,815 (15.8)	42,005 (16.4)	44,810 (15.2)
75–84	51,792 (18.1)	24,757 (18.3)	27,035 (17.9)
> =85	8942 (23.6)	4226 (22.6)	4716 (24.5)
**Education**			
< =9 years	46,205 (21.9)	20,229 (23.4)	25,976 (20.8)
> 9 years	99,621 (14.7)	50,120 (14.9)	49,501 (14.5)
Missing	1723 (23.4)	639 (25.4)	1084 (22.2)
**Equivalized household income tertile (10,000 yen/year)**			
High (247.49 < =)	51,919 (10.0)	27,298 (10.0)	24,621 (10.0)
Middle (159.1–245.97)	33,392 (16.9)	17,322 (17.9)	16,070 (15.9)
Low (<=158.77)	33,475 (25.2)	15,152 (26.8)	18,323 (23.8)
Missing	28,763 (20.5)	11,216 (22.0)	17,547 (19.5)
**Living alone**			
No	118,902 (15.2)	60,990 (15.2)	57,912 (15.2)
Yes	21,432 (26.5)	7080 (35.4)	14,352 (22.1)
Missing	7215 (19.2)	2918 (19.6)	4297 (18.9)
**Having spouse**			
Yes	107,232 (14.6)	60,203 (14.9)	47,029 (14.2)
No	37,885 (23.3)	9665 (31.5)	28,220 (20.5)
Missing	2432 (29.0)	1120 (33.3)	1312 (25.4)
**Longest occupation**			
Not farmer	126,855 (16.7)	62,189 (17.1)	64,666 (16.3)
Farmer	5378 (19.1)	2771 (19.3)	2607 (18.9)
Missing	15,316 (19.3)	6028 (19.7)	9288 (19.1)
*Area-level variables*			
**Population density quintile (person/km**^**2**^**)**			
Highest (11,759.42–37,915.64)	29,761 (17.2)	14,229 (18.0)	15,532 (16.4)
High (7431.47–11,753.88)	30,522 (16.4)	14,964 (16.9)	15,558 (15.8)
Middle (4868.92–7426.45)	29,209 (16.8)	14,155 (17.0)	15,054 (16.6)
Low (3476.89–4842.1)	29,537 (16.7)	14,094 (17.0)	15,443 (16.3)
Lowest (795.82–3456.73)	28,520 (18.4)	13,546 (18.2)	14,974 (18.5)
**Area**			
Tokai	62,932 (16.3)	30,440 (16.9)	32,492 (15.8)
Hokkaido	8486 (16.6)	4048 (16.2)	4438 (16.9)
Tohoku	7386 (20.6)	3479 (19.8)	3907 (21.4)
Hokuriku	6796 (18.7)	3314 (18.2)	3482 (19.2)
Kanto and higashiyama	35,974 (16.4)	17,651 (16.6)	18,323 (16.2)
Kinki	9426 (17.5)	4583 (19.4)	4843 (15.8)
Kyushu	16,549 (19.0)	7473 (19.4)	9076 (18.7)
**Daylight hours tertile (hours/year)**			
Short (1393.76–1886.62)	49,348 (16.9)	23,672 (17.4)	25,676 (16.3)
Middle (1886.68–2060.44)	49,797 (16.2)	24,481 (16.9)	25,316 (15.6)
Long (2060.46–2177.88)	48,404 (18.1)	22,835 (18.0)	25,569 (18.2)
Total	147,549 (17.1)	70,988 (17.4)	76,561 (16.7)
*Area-level quantitative variables (unstandardized value)*	Mean (standard deviation)	Minimum	Maximum
**Animal husbandry farm density (/1000 persons)**	0.51 (2.89)	0.00	58.30
**Crop farm density (/1000 persons)**	16.76 (82.37)	0.00	1821.40
**Amount of rain fall (mm/year)**	1533.51 (228.93)	829.75	2829.45
**Mean slope angle (%)**	7.19 (10.15)	0.00	73.11

In the null model of the multilevel Poisson regression analysis the PR of depressive symptoms varied slightly between municipalities and between neighborhoods: the MRRs were 1.13 and 1.04 in men, 1.16 and 1.00 in women, respectively (Tables 2 and 3). After incorporating explanatory variables in the model, the MRRs decreased slightly both between municipalities and between neighborhoods (Model 1 and 2), indicating that a small part of the variance in depressive symptoms between areas was explained by these variables.

**Table 2:** Prevalence ratios [95% confidence intervals] of depressive symptoms among men: the results of a multilevel analysis

	Null	Model 1		Model 2	
**Animal husbandry farm density**^a^		1.00	[0.97,1.03]	1.00	[0.97,1.03]
**Crop farm density**^a^		0.97	[0.93,1.01]	0.97	[0.93,1.01]
**Population density quintile (ref. highest)**					
High		1.00	[0.95,1.06]	1.00	[0.95,1.06]
Middle		0.99	[0.92,1.06]	0.99	[0.92,1.06]
Low		0.98	[0.91,1.06]	0.98	[0.90,1.06]
Lowest		1.04	[0.93,1.16]	1.04	[0.93,1.16]
**Area (ref. Tokai)**					
Hokkaido		0.97	[0.82,1.14]	0.97	[0.82,1.14]
Tohoku		1.20	[1.06,1.37]	1.20	[1.06,1.37]
Hokuriku		1.10	[0.98,1.22]	1.10	[0.98,1.22]
Kanto and higashiyama		1.07	[0.99,1.15]	1.07	[1.00,1.15]
Kinki		1.22	[1.11,1.34]	1.22	[1.11,1.34]
Kyushu		1.12	[0.98,1.29]	1.12	[0.98,1.29]
**Daylight hours tertile (ref. short)**					
Middle		1.00	[0.96,1.06]	1.01	[0.96,1.06]
Long		1.07	[0.99,1.15]	1.07	[0.99,1.15]
**Amount of rainfall**^a^		1.00	[0.96,1.05]	1.00	[0.96,1.05]
**Mean slope angle**^a^		0.97	[0.95,1.00]	0.97	[0.95,0.99]
*Individual-level factors*					
**Age (ref. 65–74)**					
75–84		1.04	[1.01,1.07]	1.04	[1.01,1.07]
> = 85		1.19	[1.12,1.26]	1.19	[1.12,1.26]
** Education: > 9 years (Ref. <=9 years)**		1.28	[1.24,1.32]	1.28	[1.24,1.32]
**Equivalized household income tertile (Ref. high)**					
Middle		1.65	[1.58,1.72]	1.65	[1.58,1.72]
Low		2.33	[2.21,2.46]	2.33	[2.21,2.46]
** Living alone: yes (ref. no)**		1.49	[1.40,1.58]	1.49	[1.40,1.58]
** Having spouse: no (ref: yes)**		1.42	[1.36,1.50]	1.42	[1.36,1.50]
** Longest occupation: farmer (ref: non-farmer)**		1.04	[0.95,1.14]	1.04	[0.93,1.16]
**Farmer x animal husbandry farm density**		0.96	[0.92,1.01]		
**Farmer x crop farm density**				0.97	[0.89,1.05]
*Random-effect part of the model (contextual effects)*					
Between municipality variance^b^	0.016 (0.004)	0.003 (0.002)		0.003 (0.002)	
* Median Rate Ratio*	1.13	1.05		1.05	
Between neighborhood variance^b^	0.002 (0.002)	0.000 (0.000)		0.000 (0.000)	
* Median Rate Ratio*	1.04	1.00		1.00	

^a^Per 1 standard deviation unit increase in population

^b^Standard error in parentheses

**Table 3:** Prevalence ratios [95% confidence intervals] of depressive symptoms among women: the results of a multilevel analysis

	Null	Model 1		Model 2	
**Animal husbandry farm density**^a^		1.02	[0.99,1.05]	1.02	[0.99,1.05]
**Crop farm density**^a^		0.97	[0.94,1.00]	0.97	[0.94,1.00]
**Population density quintile (ref. highest)**					
High		1.01	[0.97,1.06]	1.01	[0.97,1.06]
Middle		1.06	[1.01,1.11]	1.06	[1.01,1.11]
Low		1.04	[0.96,1.14]	1.04	[0.96,1.13]
Lowest		1.07	[0.98,1.15]	1.07	[0.98,1.15]
**Area (ref. Tokai)**					
Hokkaido		1.05	[0.85,1.28]	1.05	[0.85,1.29]
Tohoku		1.39	[1.19,1.64]	1.39	[1.19,1.63]
Hokuriku		1.19	[1.07,1.33]	1.19	[1.07,1.33]
Kanto and higashiyama		1.15	[1.06,1.25]	1.15	[1.06,1.25]
Kinki		1.11	[1.01,1.21]	1.11	[1.01,1.21]
Kyushu		1.14	[0.93,1.39]	1.14	[0.93,1.39]
**Daylight hours tertile (ref. short)**					
Middle		1.01	[0.94,1.09]	1.01	[0.94,1.09]
Long		1.06	[0.96,1.17]	1.06	[0.96,1.17]
**Amount of rainfall**^a^		1.02	[0.98,1.06]	1.02	[0.98,1.06]
**Mean slope angle**^a^		0.98	[0.96,1.00]	0.98	[0.96,1.00]
*Individual-level factors*					
**Age (ref. 65–74)**					
75–84		1.02	[0.98,1.06]	1.02	[0.98,1.06]
> = 85		1.30	[1.22,1.39]	1.30	[1.22,1.39]
** Education: > 9 years (Ref. <=9 years)**		1.24	[1.20,1.29]	1.24	[1.20,1.28]
**Equivalized household income tertile (Ref. high)**					
Middle		1.49	[1.43,1.54]	1.49	[1.43,1.55]
Low		2.09	[1.98,2.19]	2.09	[1.98,2.19]
** Living alone: yes (ref. no)**		1.13	[1.07,1.20]	1.13	[1.07,1.20]
** Having spouse: no (ref: yes)**		1.14	[1.11,1.18]	1.14	[1.11,1.18]
** Longest occupation: farmer (ref: non-farmer)**		1.07	[0.97,1.18]	1.11	[0.96,1.30]
**Farmer x animal husbandry farm density**		0.98	[0.92,1.04]		
**Farmer x crop farm density**				0.94	[0.85,1.05]
*Random-effect part of the model (contextual effects)*					
Between municipality variance^b^	0.023 (0.006)	0.006 (0.002)		0.006 (0.002)	
* Median Rate Ratio*	1.16	1.08		1.08	
Between neighborhood variance^b^	0.000 (0.000)	0.000 (0.000)		0.000 (0.000)	
* Median Rate Ratio*	1.00	1.00		1.00	

^a^Per 1 standard deviation unit increase in population

^b^Standard error in parentheses

**Fig. 1:**
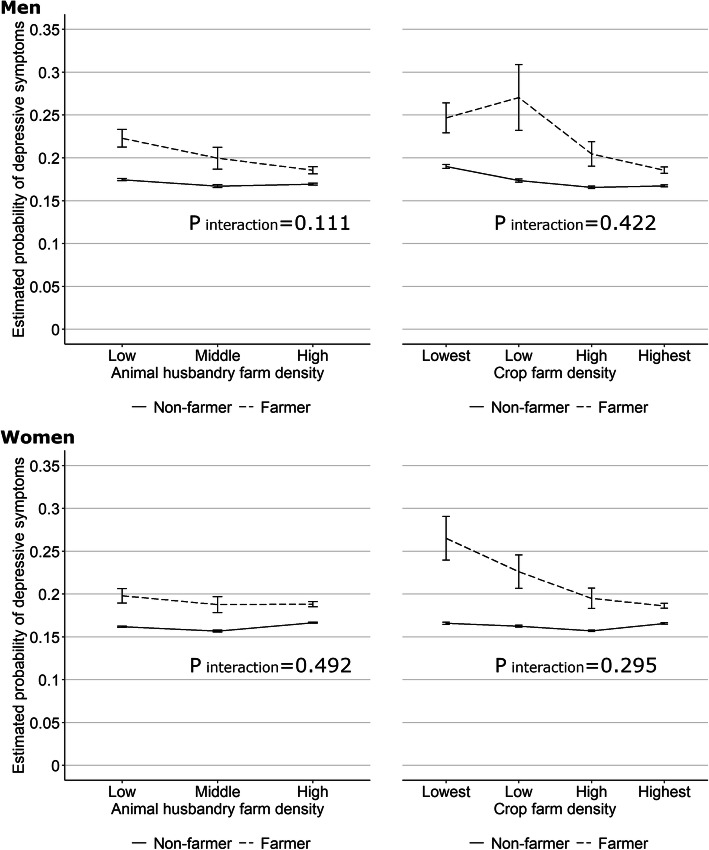
Estimated probability of depressive symptoms with 95% confidence intervals (error bars) by gender: interaction between types of agriculture in the community and longest occupation (farmer or not). The estimates were derived from a three-level multilevel Poisson regression analysis adjusted for age, education, income, living alone, marital status (having a spouse or not), animal husbandry farm density, crop farm density, daylight hours tertile, amount of rainfall, mean slope angle, population density quintile, and area

A cross-level interaction analysis showed that the probability of having depressive symptoms was higher among farmers than among non-farmers for both genders, and varied by the neighborhood farm density (Fig. 1). Among male farmers, the probability of having depressive symptoms was higher among those residing in neighborhoods where the farm density was low, compared to those in neighborhoods where the farm density was high. The estimated probability of having depressive symptoms among male farmers calculated from Model 1, which incorporated an interaction term between animal husbandry farm density and occupation, varied from 0.19 (95% confidence interval: 0.18 to 0.19) at the high animal husbandry farm density to 0.22 (0.21 to 0.23) at the low animal husbandry farm density. The estimated values calculated from Model 2, which incorporated an interaction term between crop farm density and longest occupation, varied from 0.19 (0.18 to 0.19) at the highest crop farm density to 0.25 (0.23 to 0.26) at the lowest farm density. Among female farmers, this relation was also observed for crop farm density: 0.19 (0.18 to 0.19) at the highest crop farm density to 0.27 (0.24 to 0.29) at the lowest, whereas the association was weak for animal husbandry farm density.

The *p* values for the interaction term showed that the slope of the relationship between depressive symptoms and animal husbandry farm density differed significantly between farmers and non-farmers among men (Fig. 1, the p value of the interaction term was 0.111). For crop farm density the variation in depressive risk did not differ significantly between farmers and non-farmers.

## Discussion

In this study we analyzed data from 147,549 individuals aged 65 and above residing in 1024 neighborhoods in 39 municipalities, which were collected in the 2016 wave of the Japan Gerontological Evaluation Study. Cross-level interaction analyses showed that the probability of having depressive symptoms among farmers varied by farm density with a higher risk being observed among those who were residing in neighborhoods with a low farm density, regardless of the type of agriculture. Furthermore, the variation in depressive risk according to animal husbandry farm density differed between farmers and non-farmers with a significantly higher level of depressive symptoms observed in the former. Our findings suggest that the mental health of farmers might be potentially affected by neighborhood farm density, regardless of the specific type of agriculture.

This study sheds light on the potential effects of neighborhood farm density on the risk of depressive symptoms among farmers. In particular, the finding that for farmers, the risk of depression varied by neighborhood farm density may help explain a discrepancy in previous studies about farmers’ depression. Specifically, a recent systematic review reported that although a majority of studies had found that there was a higher risk of depressive symptoms among farmers compared to in the general population, 30% of the studies nevertheless suggested that the risk was similar or even lower among farmers compared to non-farmers [1]. An earlier study conducted in a rural area in Japan showed for example, that farmers and non-farmers had a similar risk of experiencing depressive symptoms [40]. Our results may help clarify these seemingly inconsistent results as we found that the prevalence of depressive symptoms in farmers and non-farmers tends to be more similar where farm density is high, compared to where farm density is low. This suggests that it may be beneficial for future studies to examine the farming context e.g. neighborhood environmental factors such as farm density in order to better understand farmers’ mental health and the factors associated with it.

The higher prevalence of depressive symptoms among farmers in neighborhoods with a low farm density may reflect a scarcity of formal and informal social support in such communities. Farmers who live in a location where there are few other farmers may have less opportunity to talk about common concerns, such as farm management and family issues. Having access to neighbors/friends who can give and receive social support may be protective against depression [41], as well as promote access to health care [42]. Previous research has shown that a sense of belonging to a community may also affect a farmer’s mental health and wellbeing [9, 43]. In general, in Japan the farming community tends to engage in various local activities such as staging festivals and holding various events, as well as undertaking welfare activities for older adults. However, an earlier government report showed that farming communities with a small number of farms tended to have fewer of these local activities [44]. As the number of farms has declined in recent years, it is likely that the number of these local activities may have also correspondingly decreased for farmers in many parts of Japan, resulting in fewer opportunities for farmers to obtain emotional or instrumental social support from their neighbors.

It is worth noting that among men, the prevalence of depressive symptoms in relation to neighborhood animal husbandry farm density differed between farmers and non-farmers, although this result should be interpreted with caution given that there is an elevated risk of a Type I error occurring when evaluating the *p* value of interaction terms [45]. The higher risk of depressive symptoms in animal husbandry farmers compared to non-farmers in areas with few animal husbandry farms suggests that the potential effect of farm density on depressive symptoms may differ according to occupation. In particular, it is possible that animal husbandry farmers face an especially complex and potentially stressful range of responsibilities and duties [16, 46] including promoting sustainability related to the environment, ensuring animal welfare, and reducing the smell produced by livestock, which might increase the risk for poorer mental health. Moreover, community understanding of the difficulties faced by those engaging in animal husbandry might be lower where animal husbandry farming is less common. Under these circumstances those managing farms might be especially vulnerable to the effects of stress and pressure resulting from their work demands, and this may have contributed to the increased risk of having poor mental health [47]. The fact that more than 90% of farm managers in Japan are men [48] might also help explain why the cross-interaction effect was only observed among men. Furthermore, in this study, we examined farm density, which ignores the size of each farm. Even if an area contained a mega farm, it might still have been more appropriate to categorize it as having a “low” farm density. This is because animal husbandry has dramatically modernized in Japan in recent years, with a reduction in the number of farmers while the quantity of livestock has significantly increased [49]. Although we could not actually quantify the size of the individual farms in each area, these potential health effects of structural change in farm management on individual farmers and the neighborhood environment should be further researched.

### Strengths and limitations

Our study has notable strengths. It used data from a large sample in Japan, which enabled us to examine the health status of farmers, whose numbers are small relative to the overall population size. In addition, the richness of the data allowed us to use multilevel models to shed light on potential neighborhood-level factors affecting individual health. The linking mechanisms we discuss in this paper between farm density and depression might also be applicable in other countries. A focus on the health impact of neighborhood farm density, examined as a consequence of structural change in the agricultural sector, may stimulate further research as there is little evidence on this phenomenon at present.

This study also has several limitations that should be noted. First, as the dataset we used was not representative of the whole of Japan, our findings might have limited generalizability because of the coverage of the sampling frame. However, as the JAGES dataset includes residents who live in various types of urban and rural municipalities across Japan, we believe that the findings of this study are applicable to a large degree to healthy older adults nationwide. Second, for individual occupation, we could only obtain information on whether the longest occupation was farmer or not, we could not distinguish the types of agriculture each participant engaged in. For example, we could not determine whether animal husbandry farmers or crop farmers had mental health problems in neighborhoods with a low animal husbandry farm density. This may have biased the findings of our study. Third, the longest occupation variable did not include current occupational status. This means that some of the participants who answered that their longest occupation was farmer may have already stopped farming and left their farms. Future research should examine mental health differences between retired farmers and those still working. Fourth, as the data were self-reported it is possible that this may have given rise to various forms of bias such as recall bias and social desirability bias. For example, masculine norms may affect the reporting of mental health problems [8], so the higher prevalence ratios of depressive symptoms observed among male farmers might nevertheless have still been underestimated.

## Conclusions

The results of this study suggest that the mental health of individuals whose longest occupation was farmer may be potentially affected by the neighborhood farm density. The higher prevalence of depressive symptoms among farmers in neighborhoods with a low farm density may reflect a scarcity of formal and informal social support in such communities. The health effects of the neighborhood environment on farmers, such as farm density, which may vary by the type of agriculture, should be further researched.

## Data Availability

The datasets of the Japan Gerontological Evaluation Study, which were used in this research, are available from the corresponding author upon reasonable request. All enquiries should be addressed to the data management committee via e-mail: dataadmin.ml@jages.net. All JAGES datasets have ethical or legal restrictions for public deposition due to the inclusion of sensitive information from the human participants. Following the regulations of local governments which cooperated in the survey, the JAGES data management committee has imposed restrictions upon the data. The government agricultural data used in this study are openly available from the website of the Ministry of Agriculture, Forestry and Fisheries at [www.maff.go.jp/j/tokei/census/shuraku_data/2015/sa], reference number [23].

## Abbreviations

JAGES: Japan Gerontological Evaluation Study
GDS: Geriatric Depression Scale
MRR: Median Rate Ratio
